# Blastoids: a new model for human blastocyst development

**DOI:** 10.1038/s41392-021-00663-8

**Published:** 2021-06-16

**Authors:** Heiner Niemann, Bob Seamark

**Affiliations:** 1 Medizinische Hochschule Hannover (MHH), Hannover, Germany; 2 Flinders University, College of Medicine and Public Health, Adelaide, SA Australia

**Keywords:** Pluripotency

**Fig. 1:**
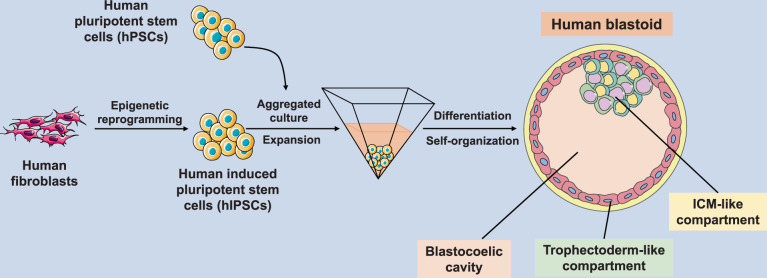
Schematic procedure for the human blastoid generation from human PSCs

Recently, two research groups report in *Nature*^1,2^ the ex-vivo production of blastocyst-like structures, called blastoids, that exhibit many of the landmarks in human early development found in viable blastocysts (Fig. 1).

The formation of a blastocyst is a critical step in early embryo development denoting a key change from the early cleavage stages to gastrulation. Typically, the blastocyst, differentiated from the early cleavage stages, is a fluid filled vesicular structure comprised of cells of now, three distinct cell lineages, namely those of the trophoblast, the outer enclosing cell layer, and those of the inner cell mass (ICM) with the hypoblast and epiblast, found in the central fluid filled cavity (the blastocoel). The formation of a viable blastocyst marks an important checkpoint in mammalian development, including the first differentiation of the pluripotent ICM from which the embryo proper is derived and the formation of the trophoblast that will later give rise to the placenta. Known accompanying changes include a switch in the metabolism of the embryo from a reliance on substrates such as pyruvate, lactate, or amino acids, to glucose uptake and oxidative metabolism needed to ensure sufficient energy supply for the developing embryo. Significantly, it is at the blastocyst stage that the telomere length is set for life.^3^

While these blastoids share important features of developmentally viable blastocysts, a number of important questions remain concerning their equivalence with their in vivo or in vitro produced counterparts.

The blastoids were derived either from naive embryonic stem cells derived from human embryos, or from induced pluripotent stem cells generated from adult fibroblasts. Advanced in vitro culture techniques involving specific 3D culture dishes and a sequential culture system were employed, with blastoids forming after 4–5 days of culture. The overall efficiency was rather low, with only ~10% of the reprogrammed cells developing to blastocyst-like structures; the efficiency varying according to cell line, derivation method, and a number of other biological and technical details. The blastoids derived exhibited many of the features of true blastocysts, including having a similar morpology, similar number of embryo-like cells and a comparable ratio of ICM and TE cells as in fertilization derived blastocysts. The blastoids also expressed typical markers of the pluripotent ICM (Oct4, Nanog) and the trophoblast (CDX2), with transcriptomic profiling revealing a mRNA expression profile that was largely similar with their fertilization derived counterparts. Moreover and importantly, under appropriate in vitro conditions, pluripotent stem cell-like cells and trophectodermal stem cells (TSC) could be derived from these blastoids. Albeit, it should be noted, that non-reprogrammed cells were regularly found within the blastoids.

While these are critical hallmarks of real blastocysts, their true developmental potential remains uncertain. Both epigenetic and chromosomal features are crucial for regular embryo development and neither has yet been sufficiently disclosed for full assesment of the blastoids. Given the specific embyo culture procedures required, the epigenetic status of the blastoids is of particular interest. Numerous studies have shown that in vitro culture of early embryos can profoundly affect normal patterns of DNA methylation, resulting in deviations from the physiological gene expression patterns.^4^ Following fertilisation, the parental genomes undergo a wave of de- and re-methylation, during early embryogenesis, creating the methylation patterns, needed for normal development, through the activation and silencing of specific genes. Typically, global methylation of the mammalian genome declines to a nadir at the 6–8 cell stage and increases thereafter; with methylation lower in female embryos than in male embryos at the blastocyst stage and lower in the ICM than TE, depending on species. The impact of the novel culture procedures used to produce the blastoids, on this wave of de-and remethylation, remains to be determined. It is also conceivable that important epigenetic features such as the genomic imprinting status are also affected by the extended culture period needed to arrive at well structured blastoids.

The critical importance of epigenetic imprints as determinants of lifetime health and fitness is well known.^5^ Genomic imprinting is a specific epigenetic phenomenon determining which allele of a specific gene is transcriptionally active through silencing the other (imprinted) according to the parent-of-origin. Approximately 50 genes are thought to be imprinted in humans. Imprinting disorders are known to be more prevalent in gametes and embryos after Assisted Reproductive Technology (ART) than in their counterparts derived from in vivo production. It is also well known, that an extended in vitro culture may be associated with deviations from the normal euploid human chromosomal set-up (46 chromosomes), leading to polyploid or aneuploid chromosomal configurations, which in turn may interfere with regular development.

Albeit whilst these blastoids denote the achievement of an important milestone in the development of a viable model for studying early human development, it is still a rudimentary model that will need significant improvement prior to becoming a broadly used research tool. For useful application, efficiency needs to be significantly increased with deviations from the fertilization derived blastocysts reduced and, hopefully, eliminated. Further assurance will also be needed on their status, in regard to their normality of chromosomal set-up, epigenetic profile, and proper lineage development, to fully realise the potential utility of such ex vivo derived blastoids in the study of early human developmental and implantation processes.

Access to human embryos is subject to tight regulation around the globe due to ethical concerns. Culture of human embryos is presently restricted to a maximum of 14 days, concomitant with the appearance of the primitive streak. The production of blastoids might lessen some of these ethical concerns allowing new avenues for basic studies into early human development. Blastocysts, grown in the laboratory from human stem cells, promise to enable studies into cell lineage formation, differentiation, early embryonic death, miscarriage, or other developmental failures. Importantly ex vivo derived blastocysts would also provide a needed model for pharmacological studies on potential effects of drugs and drug candidates on embryos and early fetuses. These and other exciting prospects warrant continuing research on human blastoids, to build on the significant achievements reported in these two papers.
